# Awareness about cervical cancer and its socio-economic determinants among adults in Bangladesh: Results from a nationwide cross-sectional study

**DOI:** 10.1371/journal.pone.0325712

**Published:** 2025-06-10

**Authors:** Tariful Islam, Md. Abdullah Saeed Khan, Mohammad Delwer Hossain Hawlader, Nur-E-Safa Meem, Fahima Nasrin Eva, Umme Kulsum Monisha, Mohammad Ali Hossain, Mohammad Hayatun Nabi

**Affiliations:** 1 Department of Public Health, North South University, Dhaka, Bangladesh; 2 Public Health Promotion and Development Society (PPDS), Dhaka, Bangladesh; 3 International Centre for Diarrhoeal Disease Research, Bangladesh (icddr, b), Mohakhali, Dhaka, Bangladesh; 4 National Institute of Preventive and Social Medicine (NIPSOM), Dhaka, Bangladesh; 5 NSU Global Health Institute (NGHI), North South University, Dhaka, Bangladesh; 6 Ibn Sina Medical College Hospital, Kallyanpur, Dhaka, Bangladesh; University of Nairobi, KENYA

## Abstract

Cervical cancer is a major global health issue now and is the second leading cancer among women in Bangladesh, caused primarily by Human papillomavirus (HPV). Despite effective vaccination and screening methods, awareness and access to preventive methods are limited in developing regions. This nationwide study aims to explore socio-economic and cultural factors affecting cervical cancer awareness in Bangladesh and generate evidence-based recommendations for tailoring suitable health promotion strategies for the country. This cross-sectional study was conducted in Bangladesh to evaluate cervical cancer awareness among adults aged 18 years and above. A semi-structured questionnaire was designed for this research, keeping in mind the local sociocultural contexts. Face-to-face interviews were conducted after getting the participants’ consent and making sure they understood all the questions. After checking for data quality and consistency, responses of 2,151 participants were finally retained. The collected data was analyzed using STATA (Version 17) statistical software. The majority (80.15%) of the participants were aware of cervical cancer, with healthcare workers being the primary source of information. Higher awareness rates were noted among married individuals (80.88%), urban dwellers (83.24%), those with more education (average 11.96 ± 4.36 years), individuals with higher income (88.17%), and those belonging to nuclear families (82.86%). Healthcare professionals had significantly greater awareness (98.91%) compared to other occupations. Participants undergoing regular health checkups were more informed and the association was statistically significant (87.03%, p < 0.001). Univariate and multiple logistic regression models revealed that each additional year of education increased the probability of being aware by 16−20%. Men had significantly lower odds of being aware compared to women (Adjusted Odds Ratio AOR = 0.41, β = −0.895, 95% CI: 0.23 to 0.70, p = 0.001 in the multiple logistic regression analysis). Similarly, respondents not cohabitating with their spouse were less aware than those who were living with partners (AOR = 0.47, β = −0.750, 95% CI: 0.28 to 0.81, p = 0.006). Income levels conversely influenced awareness level, participants from the highest income group (earning 35,001−50,000 BDT) had 47% lower odds (AOR = 0.53, β = −0.629, 95% CI: 0.36 to 0.80, p = 0.002) of awareness compared to the lowest income group (earning ≤20,000 BDT). Despite the widespread awareness of cervical cancer among most Bangladeshi adults, there remains a notable gap, particularly among certain demographics. Since the study identified healthcare workers, social media, and mass media as major sources of information, targeted educational campaigns through media channels and outreach activities by healthcare workers might effectively enhance nationwide awareness of cervical cancer.

## Introduction

Cervical cancer, predominantly caused by sexually transmitted Human papillomavirus (HPV), is a critical health issue, with a global incidence rate of 13.3 and a mortality rate of 7.3 per 100,000 women [1]. In Bangladesh, it is the second most common cancer in women, causing about 12,000 new cases and over 6,000 deaths per year [2]. The high-risk HPV strains, particularly HPV-16 and HPV-18, are responsible for over 70% of cervical cancer cases globally [3]. Vaccines against HPV, both quadrivalent (against HPV strains 6, 11, 16, and 18) and bivalent (against HPV strains 16 and 18) have shown efficacy in preventing cervical cancer [4]. Screening methods, such as the pap smear test, visual examination with acetic acid (VIA), and self-sampling for HPV DNA testing, have also made significant strides in early detection and prevention since the 1950s [5]. Incidence, morbidity, and mortality of cervical cancer vary significantly among regions and are closely interlaced with the level of awareness and accessibility to preventive measures. More than 85% of the new cases and 80% of deaths due to cervical cancer occur in low- and middle-income countries (LMICs), while there is a decline in cervical cancer incidence in developed nations due to their effective and widely adopted strategic approaches [6,7]. However, despite bearing a more significant disease burden, developing countries continue to grapple with awareness and intervention challenges.

Cervical cancer awareness varies significantly across different demographics worldwide. This challenge is especially pronounced in developing countries. In Japan, studies have shown a disparity in awareness between genders, indicating low vaccination and screening rates [8]. Research conducted in Saudi Arabia and India revealed substantial knowledge gaps about HPV and cervical cancer, along with limited awareness of preventive measures, despite the prevalence of the disease [9–11]. Similarly, a previous study in Nepal highlighted a lack of awareness about cervical cancer despite being the most common malignancy among Nepalese women [12]. The situation is mirrored in Serbia, Morocco, and Karachi, where young women, parents, and even well-educated populations showed limited knowledge and low HPV vaccine uptake [13–15]. Alarmingly, a notable gap in awareness and practices related to cervical cancer screening existed even within the medical community, as reflected in the study conducted in Karachi [16]. This underscores a universal need for comprehensive education and training. Additionally, healthcare professionals, including nurses in India and Turkey, exhibited deficiencies in awareness and practices, with some studies reporting zero HPV vaccination rates among the nurses surveyed [10,17]. Further studies from Nepal, Cyprus, and India revealed that socio-economic conditions, cultural beliefs, healthcare trust, and fear of postvaccination complications affected vaccination awareness and preventive measures [18–20].

South Asian countries, including Bangladesh, bear nearly one-third of the global cervical cancer burden [21]. Despite the seriousness of the situation, population-based screening initiatives are conspicuously absent, and public unawareness forms a formidable barrier. Obstacles such as the high cost of vaccines are impeding the effective implementation of preventive strategies for cervical cancer. Studies illuminated concerning gaps in knowledge about cervical cancer among Bangladeshi women, only 12% of women in Bangladesh were aware of cervical cancer screening [22]. Deeply rooted socio-cultural norms like early marriage contributed to the higher prevalence of cervical cancer in Bangladesh [23].

Given the urgency and gravity of the issue, this nationwide study on the awareness of cervical cancer among adults in Bangladesh aims to provide deeper insights, considering various socio-economic, cultural, and educational factors. As the existing level of awareness regarding prevention, screening, and vaccination is fragmented, this study aims to investigate the real and nationwide scenario on the awareness level. This research was also designed to identify the need for comprehensive policies and specific awareness campaigns tailored to the unique sociocultural context of Bangladesh.

## Materials and methods

### Geographic scope

The study was conducted across Bangladesh, a South Asian country known for its rich and varied culture. The country is divided into eight administrative divisions, which consist of 64 districts, 495 Upazilas, and 4571 unions [24]. Considering the accessibility, this study covered 42 of these districts across the divisions, providing a comprehensive representation of the country’s geographic and cultural diversity.

### Study design and participants

A cross-sectional study was conducted among citizens of Bangladesh including both males and females who were at least 18 years old at the time of data collection. The study employed convenience sampling method and covered all eight administrative divisions of the country: Dhaka, Mymensingh, Chattogram, Sylhet, Rajshahi, Khulna, Rangpur, and Barisal. The data was collected between June 28th and August 16th, 2023.

### Study participants and inclusion-exclusion criteria

**Fig 1:**
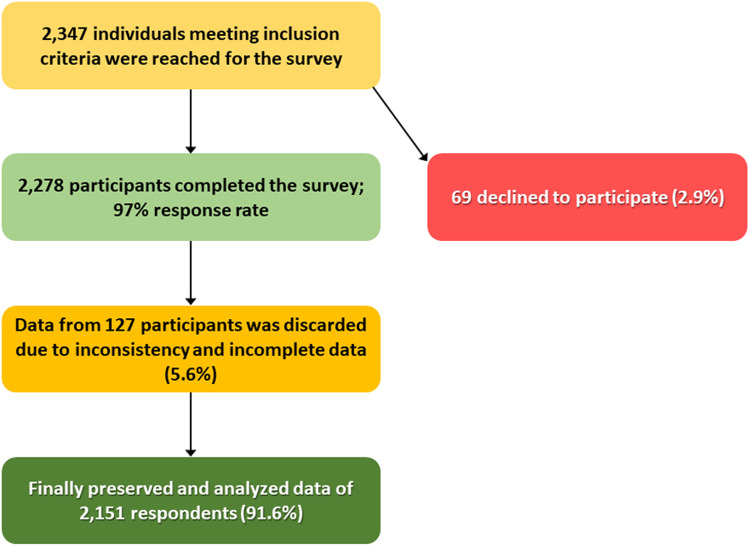
CONSORT flow chart of study participants.

The research was tailored to include individuals who were permanent residents of Bangladesh, possessed a government-issued national identity card (NID), and were willing to participate. People with mental illnesses, dual citizenship, or those who were unwilling to participate were excluded from the study. Considering the 71.8% awareness level among 600 Bangladeshi rural women identified in a prior study [2], the sample size was determined based on the formula for unknown population [25], resulting in a total adjusted sample size of 2,149 participants, with the distribution reflecting the demographics of the eight divisions according to the 2022 population & housing census (Mymensingh and Sylhet divisions were considered as single unit during sample size calculation considering the area, number of districts and population size) [26]. After the cleaning process, data from 2,151 adult participants from Bangladesh meeting the inclusion criteria was kept for final analysis (Fig 1).

### Data collection instrument

During the study, a semi-structured questionnaire was used to conduct face-to-face interviews with participants to determine their level of awareness about cervical cancer. Initially, a draft English version of the questionnaire was created based on previous research after completing a thorough literature review [2,16,21–23,27–36]. The phrasing of the questions was further changed to better align with Bangladesh’s sociocultural and healthcare contexts. After modifying the questionnaire, it was translated into Bangla and verified through consensus among researchers with multiple revisions. Due to the urgency of the study, certified translators’ forward-background translation and relevant experts’ assessments were not included. However, the questionnaire’s face validity was ensured through expert opinions. Changes were made to the questionnaire items to enhance face and construct validity, making it easier for participants to understand. Finally, individuals from diverse socioeconomic backgrounds were selected for the pre-testing and participants' feedback was incorporated into the survey to maintain consistency with contemporary research. The final questionnaire consisted of 17 items in two domains: socio-demographic variables (15 items) and awareness about cervical cancer (2 items). The English version of the questionnaire is available as supplementary material S1 File.

### Data collection procedure

A team of trained public health graduates and students collected data for the study. The interviewers engaged with a wide array of participants without considering their backgrounds. Interviewers were strategically assigned based on their local areas to overcome any potential language barriers. Participants were recruited in various public and private settings, including hospitals, schools, pharmacies, markets, streets, workplaces, and residences. Beyond these, the team also reached out to their personal contacts, including family, friends, neighbors, and colleagues. To ensure clarity and understanding, interviewers explained the more complex questions to the participants for their better understanding while they were filling out the questionnaire. All study participants provided written informed consent, which was obtained voluntarily before their inclusion in the study.

### Variables of interest

#### Independent variables.

The study evaluated several variables to determine the level of awareness about cervical cancer among Bangladeshi nationals and its association with other socio-demographic factors. Personal information about the participants was collected, including their address (division and district), age, gender, marital status, religion, place of residence, educational background in years, occupation, whether they work in healthcare, average monthly family income, family size, family type, and how often they have routine health checkups. Yes/no questions were used to determine healthcare worker status. The participants’ residential locations were classified as rural, semi-urban, or urban. The frequency of health checkups was also assessed and grouped into three categories: regular, irregular, and never. Specific time intervals were used, such as “<1 year,” “1-2 years,” “2-5 years,” and “>5 years,” to provide a comprehensive overview of participants’ health monitoring practices.

#### Dependent variables.

The survey used a single closed-ended question to assess the participants’ awareness of cervical cancer, which required a “yes” or “no” response. If the participants answered “yes,” they were asked to specify the source(s) of their information on the subject in a dichotomous (yes/no) response.

### Data analysis

Before conducting the analysis, the collected data was thoroughly checked for completeness, outliers, and any violations of the underlying assumptions. Descriptive statistics were used to describe the socio-demographic characteristics of the participants, as well as their awareness and sources of information about cervical cancer. The relationship between the participants’ characteristics and awareness of cervical carcinoma (binomial factor variable) was assessed using multiple tests, including Pearson’s Chi-squared test and Fisher’s Exact tests for nominal factor variable, Welch’s Two Sample t-test for parametric continuous factor variable and the Wilcoxon Rank Sum test for non-parametric continuous factor variable. The normality of the continuous data was checked using histogram and normal curve, and Shapiro-Wilk test. Univariate and multiple logistic regression analyses were performed to investigate the independent association between individuals’ awareness of cervical cancer, their socio-demographic factors, and the frequency of their health checkups. Factors that came significant at p < 0.2 level in the univariate analysis were considered for the multiple logistic regression analysis. This approach allowed for broader inclusion of variables to detect their impact when considered combinedly in the multiple logistic regression model [37]. Thus, by incorporating more variables, it was ensured to capture those variables that could have been missed if a lower cut-off point for p-value was considered [38]. The results were presented as adjusted odds ratios (AOR) with coefficient (β), standard error (SE) of β, and 95% confidence intervals (CI) for multiple logistic regression analysis. A p-value of less than 0.05 was considered statistically significant for all tests conducted. The statistical software STATA, Version 17, was used for the analysis.

### Ethical consideration

The study followed ethical standards set by the Institutional Review Board (IRB) and Ethical Review Committee (ERC) of the North South University. The approval for the study was granted under the number 2023/OR-NSU/IRB/0507. The study adhered to the ethical norms outlined in the 1964 Declaration of Helsinki and its subsequent amendments or similar ethical standards where applicable [39]. All participants provided written informed consent during in-person interviews and were informed before starting the interview of their right to withdraw at any time. Participants were also assured that their data would be accessed only by the research team and reported in aggregate form.

## Result

**Fig 2:**
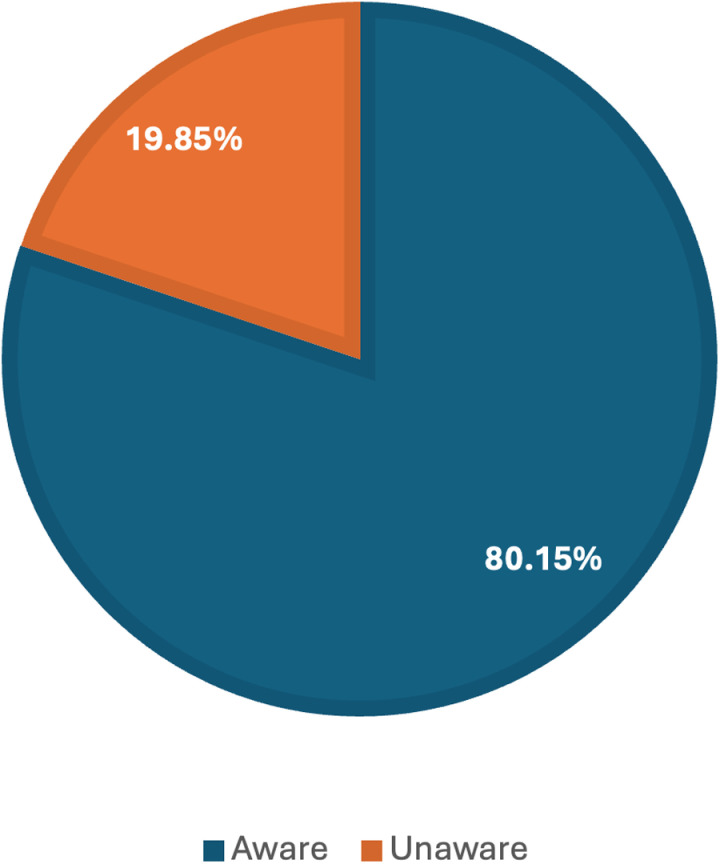
Status of awareness about cervical cancer among participants.

This nationwide study aims to assess the participants’ awareness of cervical cancer. According to the data obtained, most participants, precisely 80.15%, demonstrated awareness of cervical cancer (Fig 2). However, a smaller proportion of individuals (19.85%) were unaware of the condition. This highlights that while a majority of the participants are informed about cervical cancer, there still exists a significant minority that lacks essential knowledge about the disease.

Table 1 illustrates the level of awareness regarding cervical cancer and its socio-economic determinants among adults in Bangladesh. This study was conducted nationwide and involved 2,151 participants, with an average age of 38.18 years and a standard deviation of 5.86 years. Of the total, 81.40% (n = 1,751) were females, while 18.60% (n = 400) were males. The majority of the participants, 93.86% (n = 2,019), reported living with their spouse. In contrast, 3.53% (n = 76) were living without a spouse, and 2.60% (n = 56) were categorized as others, which included divorced, separated, or widowed individuals. A significant proportion of the participants, 82.94% (n = 1,784), were followers of Islam, while 15.67% (n = 337) were Hindus. Buddhists and Christians comprised small percentage of the total number of respondents, accounting for only 0.19% (n = 4) and 1.21% (n = 26), respectively. Urban participants were more prevalent at 51.60% (n = 1,110), followed by rural participants at 36.40% (n = 783) and semi-urban participants at 11.99% (n = 258). On an average, the participants had 11.37 years of education, with a standard deviation of 4.51 years. 23.76% (n = 511) of the participants were engaged in formal jobs, while 8.65% (n = 186) were involved in business. The majority of the female participants, 63.41% (n = 1,364), were identified as housewives. 8.51% (n = 183) of the participants were healthcare workers. The study participants were recruited from different socio-economic classes, with 25.47% (n = 547) earning BDT 20,000 or less, 26.49% (n = 569) earning between BDT 20,001 and BDT 35,000, 27.98% (n = 601) earning from BDT 35,001 to BDT 50,000, and 20.07% (n = 431) earning more than BDT 50,000. The median number of family members was five, with an interquartile range from four to six. 42.88% (n = 922) of the participants were from nuclear families, while 57.12% (n = 1,228) belonged to joint families. A total of 31.19% (n = 671) of the participants received regular health checkups, 41.38% (n = 890) had irregular checkups, and 27.43% (n = 590) had never undergone a health checkup.

**Table 1:** Characteristics of the participants (n = 2,151).

Characteristic	Statistics
**Age (years)**	38.18 ± 5.86^1^
**Sex**	
Female	1,751 (81.40)^2^
Male	400 (18.60)^2^
**Marital Status**	
Living with spouse	2,019 (93.86)^2^
Living without spouse	76 (3.53)^2^
Others	56 (2.60)^2^
**Religion**	
Islam	1,784 (82.94)^2^
Hindu	337 (15.67)^2^
Buddhist	4 (0.19)^2^
Christian	26 (1.21)^2^
**Residence**	
Rural	783 (36.40)^2^
Semi-urban	258 (11.99)^2^
Urban	1,110 (51.60)^2^
**Years of Education**	11.37 ± 4.51^1^
**Occupation**	
Job	511 (23.76)^2^
Business	186 (8.65)^2^
Housewife	1,364 (63.41)^2^
Others	90 (4.18)^2^
**Health Care Worker**	183 (8.51)^2^
**Monthly household income (BDT)**	
<=20000	547 (25.47)^2^
20001–35000	569 (26.49)^2^
35001–50000	601 (27.98)^2^
>50000	431 (20.07)^2^
**Number of family members**	5.00 (4.00, 6.00)^3^
**Family Type**	
Nuclear	922 (42.88)^2^
Joint	1,228 (57.12)^2^
**Routine Health Checkup**	
Regular	671 (31.19)^2^
Irregular	890 (41.38)^2^
Never	590 (27.43)^2^

^1^Mean ±SD; ^2^n (%); ^3^Median (IQR).

Table 2 outlines the sources of information regarding cervical cancer among the participants who were aware of the disease. Multiple responses were taken in case of information source. A significant majority, 67.59% (n = 999), reported hearing about cervical cancer from healthcare workers. In contrast, a smaller percentage, 10.29% (n = 114), gained awareness about cervical cancer through training. Friends served as a source of information for 34.71% (n = 396) of the respondents, while 17.32% (n = 193) became aware of cervical cancer through advertisements. Notably, a significant percentage of 40.96% (n = 530) mentioned that they had heard about cervical cancer via social media platforms. Additionally, mass media, including newspapers, television, and radio, was the source of information for 46.67% (n = 637) of the participants, while an equal percentage of 46.67% (n = 637) reported pharmacists as their source of information. Schools played a role in the awareness for 11.10% (n = 120) of the participants. These varied sources demonstrate the diverse channels through which individuals in the study population learned about cervical cancer.

**Table 2:** Source of information regarding cervical cancer among aware participants.

Characteristic	n (%)
Heard about cervical cancer from health care workers	999 (67.59)
Heard about cervical cancer from training	114 (10.29)
Heard about cervical cancer from friends	396 (34.71)
Heard about cervical cancer from advertisement	193 (17.32)
Heard about cervical cancer from social media	530 (40.96)
Heard about cervical cancer from mass media	637 (46.67)
Heard about cervical cancer from pharmacists	637 (46.67)
Heard about cervical cancer from school	120 (11.10)

Table 3 provides an overview of the relationship between the characteristics of the study participants and their presence of awareness about cervical cancer. The average age of participants unaware of cervical cancer was 38.63 ± 6.43 years, while for those who were aware, it was 38.07 ± 5.70 years (p-value = 0.098). Approximately 19.07% (n = 334) of females were unaware of cervical cancer and 80.93% (n = 1,417) were aware. In comparison, 23.25% (n = 93) and 76.75% (n = 307) males were unaware and aware (p = 0.059), respectively. Among the participants living with a spouse, only 19.12% were unaware of cervical cancer, while 80.88% were aware. However, 38.16% of participants residing without a spouse were unaware, with 61.84% showing awareness (p < 0.001). The prevalence of awareness was 74.84%, 82.95% and 83.24% among rural, semi-urban and urban dwellers, respectively (p < 0.001). Participants unaware of cervical cancer had an average of 9.01 ± 4.34 years of education, while those who were aware had a higher average duration (11.96 ± 4.36 years) of education (p < 0.001). The study findings revealed that 93.54% of those having a job were aware about cervical cancer and the proportion was 67.74% and 77.42% among businessmen and homemakers, respectively (p < 0.001). However, only 1.09% of the healthcare workers were unaware of cervical cancer. Awareness about cervical cancer was higher among those having monthly income above 35,000 BDT (p < 0.001). The median family size for the unaware group was 5 (IQR: 4–6), and for the aware group, it was also 5 (IQR: 4–5) (p = 0.002). Among participants from nuclear and joint families, 82.86% and 78.09% were aware about cervical cancer, respectively (p = 0.006). The population undergoing regular health checkups had higher awareness about cervical cancer compared to those either irregularly or never checked their health (p < 0.001).

**Table 3:** Association of characteristics of the participants with cervical cancer awareness.

Characteristic	Aware about cervical cancer^1^		p-value
	No (n = 427)	Yes (n = 1,724)	
**Age (years)**	38.63 ± 6.43	38.07 ± 5.70	0.098^2^
**Sex**			0.059^3^
Female	334 (19.07)	1,417 (80.93)	
Male	93 (23.25)	307 (76.75)	
**Marital Status**			**<0.001** ^3^
Living with spouse	386 (19.12)	1,633 (80.88)	
Living without spouse	29 (38.16)	47 (61.84)	
Others	12 (21.43)	44 (78.57)	
**Religion**			**<0.001** ^4^
Islam	379 (21.24)	1,405 (78.76)	
Hindu	39 (11.57)	298 (88.43)	
Buddhist	1 (25.00)	3 (75.00)	
Christian	8 (30.77)	18 (69.23)	
**Residence**			**<0.001** ^3^
Rural	197 (25.16)	586 (74.84)	
Semi-urban	44 (17.05)	214 (82.95)	
Urban	186 (16.76)	924 (83.24)	
**Years of Education**	9.01 ± 4.34	11.96 ± 4.36	**<0.001** ^2^
**Occupation**			**<0.001** ^3^
Job	33 (6.46)	478 (93.54)	
Business	60 (32.26)	126 (67.74)	
Housewife	308 (22.58)	1,056 (77.42)	
Others	26 (28.89)	64 (71.11)	
**Health Care Worker**	2 (1.09)	181 (98.91)	**<0.001** ^3^
**Monthly household income (BDT)**			**<0.001** ^3^
<=20000	126 (23.03)	421 (76.97)	
20001–35000	141 (24.78)	428 (75.22)	
35001–50000	108 (17.97)	493 (82.03)	
>50000	51 (11.83)	380 (88.17)	
**Number of family members**	5.00 (4.00, 6.00)	5.00 (4.00, 5.00)	**0.002** ^5^
**Family Type**			**0.006** ^3^
Nuclear	158 (17.14)	764 (82.86)	
Joint	269 (21.91)	959 (78.09)	
**Routine Health Checkup**			**<0.001** ^3^
Regular	87 (12.97)	584 (87.03)	
Irregular	220 (24.72)	670 (75.28)	
Never	120 (20.34)	470 (79.66)	

^1^Mean ±SD; n (%); Median (IQR).

^2^Indepedent Samples t-test.

^3^Pearson’s Chi-squared test.

^4^Fisher’s exact test.

^5^Wilcoxon rank sum test.

Table 4 presents the findings on factors impacting participants’ cervical cancer awareness using multiple logistic regression analysis. Males had significantly lower odds of being aware of cervical cancer than females, with a 59% decrease (AOR = 0.41, β = −0.895, 95% CI: 0.23 to 0.70, p < 0.001). Participants living without a spouse had significantly lower odds of awareness, with a 53% decrease (AOR = 0.47, β = −0.750, 95% CI: 0.28 to 0.81, p = 0.006). Education was found to be positively associated with awareness. For each additional year of education, the odds of being aware of cervical cancer increased by 19% (AOR = 1.19, β = 0.176, 95% CI: 1.15 to 1.24, p < 0.001). In terms of occupation, individuals in business, being a housewife, or in other professions had significantly lower odds of being aware, with businesspersons having a 61% decrease (AOR = 0.39, β = −0.935, 95% CI: 0.23 to 0.65, p < 0.001), housewives having a 63% decrease (AOR = 0.37, β = −1.00, 95% CI: 0.21 to 0.62, p < 0.001), and others having a 60% decrease (AOR = 0.40, β = −0.927, 95% CI: 0.20 to 0.78, p = 0.007). Healthcare workers had significantly higher odds of being aware compared to non-healthcare workers (AOR = 9.38, β = 2.240, 95% CI: 2.85 to 57.9, p = 0.002). Income was inversely associated with awareness, with participants earning 20,001−35,000 BDT having 46% lower odds (AOR = 0.54, β = −0.625, 95% CI: 0.39 to 0.74, p < 0.001) and those earning 35,001−50,000 BDT having 47% lower odds (AOR = 0.53, β = −0.629, 95% CI: 0.36 to 0.80, p = 0.002) compared to those earning ≤20,000 BDT. Lastly, participants with irregular health checkups had 40% lower odds of awareness (AOR = 0.60, β = −0.507, 95% CI: 0.45 to 0.81, p < 0.001).

**Table 4:** Multiple logistic regression analysis showing factors associated with cervical cancer awareness among participants.

Characteristic	β	SE of β	AOR	95% CI	p-value
**Age (years)**	−0.012	0.012	0.99	0.97 to 1.01	0.317
**Sex**					
Female	—	—	—	—	
Male	−0.895	0.278	0.41	0.23 to 0.70	**0.001**
**Marital Status**					
Living with spouse	—	—	—	—	
Living without spouse	−0.750	0.272	0.47	0.28 to 0.81	**0.006**
Others	−0.054	0.384	0.95	0.46 to 2.08	0.889
**Residence**					
Rural	—	—	—	—	
Semi-urban	0.005	0.209	1.00	0.67 to 1.52	0.982
Urban	−0.244	0.170	0.78	0.56 to 1.10	0.153
**Years of Education**	0.176	0.020	1.19	1.15 to 1.24	**<0.001**
**Occupation**					
Job	—	—	—	—	
Business	−0.935	0.263	0.39	0.23 to 0.65	**<0.001**
Housewife	−1.00	0.275	0.37	0.21 to 0.62	**<0.001**
Others	−0.927	0.345	0.40	0.20 to 0.78	**0.007**
**Health Care Worker**					
No	—	—	—	—	
Yes	2.240	0.729	9.38	2.85 to 57.9	**0.002**
**Monthly household income (BDT)**					
<=20000	—	—	—	—	
20001–35000	−0.625	0.164	0.54	0.39 to 0.74	**<0.001**
35001–50000	−0.629	0.206	0.53	0.36 to 0.80	**0.002**
>50000	−0.447	0.250	0.64	0.39 to 1.04	0.073
**Family Type**					
Nuclear	—	—	—	—	
Joint	0.245	0.131	1.28	0.99 to 1.65	0.062
**Routine Health Checkup**					
Regular	—	—	—	—	
Irregular	−0.507	0.152	0.60	0.45 to 0.81	**<0.001**
Never	−0.228	0.116	0.80	0.57 to 1.10	0.169

β: Coefficient; AOR: Adjusted odds Ratio; CI: Confidence Interval; SE: Standard Error.

## Discussion

This nationwide survey provides a comprehensive understanding of cervical cancer awareness and its socio-economic determinants among adults in Bangladesh. The majority of the participants demonstrated awareness of cervical cancer, which is consistent with findings from previous studies [2,20,31,33]. However, the persistence of a 19.85% unaware minority underscores the need for targeted interventions to address this knowledge gap.

The results of this study indicate a higher awareness level among females compared to males, although this difference is not statistically significant. This observation aligns with global trends identified in previous studies [8,31,40] and may be attributed to the direct impact cervical cancer has on women’s health. This result indicates the existing gender-based disparities in health awareness that warrant further attention. Notably, marital status seems to play a significant role in the level of awareness, with individuals living with spouses being more informed. A study by Rajon Banik et al. [2] similarly found that married women were more aware of cervical cancer (76.7%) compared to single women (69.0%). This could be due to the shared responsibilities for health between spouses in family settings, resulting in more exposure to healthcare information. The predominance of housewives among female participants (77.9%) and the study participants largely being female (81.4%) in this study might influence this inference. However, those without spouses, including divorced, separated, or widowed, exhibited lower awareness levels. This trend highlights a potential gap in health education outreach programs for these groups.

The significant association between religion and awareness highlights the role of cultural practices and community structures in the dissemination of health information. This corresponds with the research findings by S M Shahida et al. [23]. Participants from the Hindu community demonstrated the highest level of awareness in this study. The differences in awareness levels across various religions may reflect varying degrees of engagement with health education within these communities. Urban residents showed higher awareness levels compared to rural counterparts, consistent with studies highlighting urban-rural disparities in health communication [29,41]. This indicates that multisectoral and diversified communication strategies are required to resolve the urban-rural contrasts. The study findings illustrate that education is one of the key determinants in raising health awareness, further supported by prior studies [2,12,14,29,32,42–44]. This underscores the importance of educational initiatives as a medium for health promotion.

Occupation emerged as a significant determinant with healthcare workers being the most informed group, while homemakers and business professionals demonstrated lower awareness levels. This indicates a gap in the effective dissemination of health messages and a lack of health-related communication in non-healthcare environments. In a study among women of reproductive age in Southwest Ethiopia, Keneni Chali et al. [45] discovered that employed women had significantly better knowledge (60%) about cervical cancer compared to homemakers and merchants (25.3% and 20.4%, respectively). Income levels positively influence awareness, suggesting better access to resources among higher-income groups, including health information and services. Though a contrasting result was found by Guzhalinuer Abulizi et al. [44] in their study among Uyghur women from Xinjiang, China, this association suggests that economic barriers may limit health awareness highlighting the need for affordable health education initiatives.

Family structure significantly influences healthcare awareness, with nuclear families demonstrating a higher awareness level than joint families. This disparity might be attributed to more effective resource allocation for healthcare and closer bonds among the members of nuclear families. Research conducted by Neha Tripathi et al. supports this observation, they discovered that nuclear families had more intermediate to high levels of awareness (73.11% and 7.56%, respectively) than joint families (69.75% and 5.56%, respectively) [46]. The significant association between regular health checkups and awareness demonstrates the role of the healthcare system in promoting awareness. A similar positive association was identified in a previous study by Choi YJ et al. [47]. This result also underscores the need for more inclusive health services that reach out to those who do not regularly access the healthcare system.

The findings of this nationwide cross-sectional study are crucial for guiding public health strategies aimed at increasing cervical cancer awareness in Bangladesh. The high impact of healthcare workers in disseminating information underscores the importance of strengthening their role in educational campaigns. Moreover, the influence of social media and mass media indicates that these platforms might effectively facilitate health education. Free or low-cost initiatives targeting socio-economically disadvantaged groups might be beneficial in reducing disparities in cervical cancer awareness. Further studies are required to generate evidence and assess the impact of such intervention.

### Strengths and limitations

The strengths of this study make it a valuable contribution to raising cervical cancer awareness. This study comprehensively analyzed socio-demographic determinants impacting cervical cancer awareness in Bangladesh. The study was conducted across various geographic and socio-cultural contexts, including a diverse range of Bangladeshi nationals from different beliefs and socioeconomic backgrounds throughout the country. Recruitment of a representative study sample from all over Bangladesh is a significant strength of this study, which enhanced the findings’ generalizability. Moreover, the nationwide survey design of the study allowed for the collection of robust and reliable data. These strengths offer crucial insights for the development of targeted public health campaigns and interventions aimed at enhancing awareness of cervical cancer, ensuring that the study is relevant not only in Bangladesh but also in similar socio-demographic contexts.

However, the study is not devoid of limitations. As the study was cross-sectional in nature, no causal relationship could be established. Additionally, the use of self-reported data may cause potential biases. Convenience sampling may also create selection bias, affecting the study’s overall representativeness. During the house-to-house visit, the data collectors found the housewives in most of the cases (63.41%) to respond as the male counterparts usually remained outside during working hours. The absence of longitudinal data also prevents the examination of awareness trends. Moreover, the study primarily provides quantitative insights without any qualitative depth, to explore the underlying reasons for socio-demographic determinants of cervical cancer awareness. These factors should be evaluated when interpreting the findings and should be considered in future studies.

## Conclusion

This nationwide cross-sectional study has effectively mapped the landscape of cervical cancer awareness in Bangladesh. The findings have revealed that most adults are well-informed, which aligns with previous research. However, there are still a number of populations lacking awareness of this preventable cancer, emphasizing the need for tailored educational programs. The study has identified the impact of factors like marital status, religion, geographic location, education, occupation, income, and family structure on cervical cancer awareness. These results underscore the multifaceted nature of health awareness and recommend strategic public health interventions illustrating its necessity. Strengthening the outreach efforts of healthcare workers, particularly at the community level, and utilizing the power of social and mass media might enhance health education and services, making them more inclusive and accessible. These initiatives may ultimately improve public awareness of cervical cancer across the nation. Future interventional studies might provide evidence-based insight into this.

## Supporting information

S1 File English version of the questionnaire. (DOCX)

S2 Table Supplementary table showing univariate logistic regression analysis. (DOCX)

## References

[1] Gultekin M, Ramirez PT, Broutet N, Hutubessy R. World Health Organization call for action to eliminate cervical cancer globally. Int J Gynecol Cancer. 2020;30(4):426–7. doi: 10.1136/ijgc-2020-001285 32122950

[2] Banik R, Naher S, Rahman M, Gozal D. Investigating Bangladeshi Rural Women’s Awareness and Knowledge of Cervical Cancer and Attitude Towards HPV Vaccination: a Community-Based Cross-Sectional Analysis. J Cancer Educ. 2022;37(2):449–60. doi: 10.1007/s13187-020-01835-w 32734448

[3] Dereje N, Ashenafi A, Abera A, Melaku E, Yirgashewa K, Yitna M, et al. Knowledge and acceptance of HPV vaccination and its associated factors among parents of daughters in Addis Ababa, Ethiopia: a community-based cross-sectional study. Infect Agent Cancer. 2021;16(1):58. doi: 10.1186/s13027-021-00399-8 34479576 PMC8418033

[4] Kilic A, Seven M, Guvenc G, Akyuz A, Ciftci S. Acceptance of human papillomavirus vaccine by adolescent girls and their parents in Turkey. Asian Pac J Cancer Prev. 2012;13(9):4267–72. doi: 10.7314/apjcp.2012.13.9.4267 23167326

[5] Spayne J, Hesketh T. Estimate of global human papillomavirus vaccination coverage: analysis of country-level indicators. BMJ Open. 2021;11(9):e052016. doi: 10.1136/bmjopen-2021-052016 34475188 PMC8413939

[6] LaVigne AW, Triedman SA, Randall TC, Trimble EL, Viswanathan AN. Cervical cancer in low and middle income countries: Addressing barriers to radiotherapy delivery. Gynecol Oncol Rep. 2017;22:16–20. doi: 10.1016/j.gore.2017.08.004 28948205 PMC5602511

[7] Beddoe AM. Elimination of cervical cancer: challenges for developing countries. Ecancermedicalscience. 2019;13:975. doi: 10.3332/ecancer.2019.975 31921346 PMC6946419

[8] Horio F, Ikeda T, Zaitsu M, Takebe D, Tabata A, Matsukura M, et al. Knowledge and Awareness of Human Papillomavirus Vaccination and Cervical Cancer among Men and Women in Japan: A Questionnaire Survey. Asian Pac J Cancer Prev. 2023;24(3):1063–71. doi: 10.31557/APJCP.2023.24.3.1063 36974562 PMC10334079

[9] Almehmadi MM, Salih MM, Al-Hazmi AS. Awareness of human papillomavirus infection complications, cervical cancer, and vaccine among the Saudi population. A cross-sectional survey. Saudi Med J. 2019;40(6):555–9. doi: 10.15537/smj.2019.6.24208 31219489 PMC6778758

[10] Jain SM, Bagde MN, Bagde ND. Awareness of cervical cancer and Pap smear among nursing staff at a rural tertiary care hospital in Central India. Indian J Cancer. 2016;53(1):63–6. doi: 10.4103/0019-509X.180823 27146744

[11] Pandey S, Chandravati. Human Papillomavirus-mediated cervical cancer awareness and Gardasil vaccination: a pilot survey among North Indian women. J Community Health. 2013;38(5):907–10. doi: 10.1007/s10900-013-9697-6 23653161

[12] Thapa M. Cervical Cancer Awareness and Practice of Pap Smear Test Among Women with Gynecological problems. JNMA J Nepal Med Assoc. 2018;56(211):654–7. doi: 10.31729/jnma.3633 30381758 PMC8997268

[13] Rančić NK, Golubović MB, Ilić MV, Ignjatović AS, Živadinović RM, Đenić SN, et al. Knowledge about Cervical Cancer and Awareness of Human Papillomavirus (HPV) and HPV Vaccine among Female Students from Serbia. Medicina (Kaunas). 2020;56(8):406. doi: 10.3390/medicina56080406 32823648 PMC7466248

[14] Mouallif M, Bowyer HL, Festali S, Albert A, Filali-Zegzouti Y, Guenin S, et al. Cervical cancer and HPV: Awareness and vaccine acceptability among parents in Morocco. Vaccine. 2014;32(3):409–16. doi: 10.1016/j.vaccine.2013.10.069 24188754

[15] Hirani S, Khan S, Akram S, Virji SN, Shaikh PA, Naeem E, et al. Knowledge, awareness, and practices of cervical cancer, its risk factors, screening, and prevention among women in Karachi, Pakistan. Eur J Cancer Prev. 2021;30(1):97–102. doi: 10.1097/CEJ.0000000000000590 32301762

[16] Majid E, Shaikh MA, Qazi OA, Khan S, Majeed I, Bano K. Awareness, screening, practices and attitudes of cervical cancer among doctors and nursing staff working at a tertiary care centre. J Pak Med Assoc. 2022;72(6):1025–30. doi: 10.47391/JPMA.1443 35751302

[17] Koç Z, Çinarli T. Cervical Cancer, Human Papillomavirus, and Vaccination. Nursing Research. 2015;64(6):452–65. doi: 10.1097/nnr.0000000000000125 26505158

[18] Bhatta MP, Johnson DC, Lama M, Maharjan B, Lhaki P, Shrestha S. Cervical Cancer and Human Papillomavirus Vaccine Awareness Among Married Bhutanese Refugee and Nepali Women in Eastern Nepal. J Community Health. 2020;45(3):516–25. doi: 10.1007/s10900-019-00770-2 31696420

[19] Farazi PA, Siahpush M, Michaud TL, Kim J, Muchena C. Awareness of HPV and Cervical Cancer Prevention Among University Health Sciences Students in Cyprus. J Cancer Educ. 2019;34(4):685–90. doi: 10.1007/s13187-018-1356-2 29629509

[20] Singh J, Roy B, Yadav A, Siddiqui S, Setia A, Ramesh R, et al. Cervical cancer awareness and HPV vaccine acceptability among females in Delhi: A cross-sectional study. Indian J Cancer. 2018;55(3):233–7. doi: 10.4103/ijc.IJC_28_18 30693885

[21] Sankaranarayanan R, Bhatla N, Gravitt PE, Basu P, Esmy PO, Ashrafunnessa KS, et al. Human papillomavirus infection and cervical cancer prevention in India, Bangladesh, Sri Lanka and Nepal. Vaccine. 2008;26 Suppl 12:M43-52. doi: 10.1016/j.vaccine.2008.05.005 18945413

[22] Ferdous J, Islam S, Marzen T. Attitude and practice of cervical cancer screening among the women of Bangladesh. Mymensingh Med J. 2014;23(4):695–702. 25481587

[23] Shahida SM, Saha K, Banu KA, Islam MA. Camp based awareness and screening programme of cervical cancer in rural area of Bangladesh. Mymensingh Med J. 2013;22(4):640–5. 24292289

[24] Divisions of Bangladesh. Wikipedia. 2023. https://en.wikipedia.org/w/index.php?title=Divisions_of_Bangladesh&oldid=1176358600

[25] Charan J, Biswas T. How to calculate sample size for different study designs in medical research?. Indian J Psychol Med. 2013;35(2):121–6. doi: 10.4103/0253-7176.116232 24049221 PMC3775042

[26] Population & Housing Census: Preliminary Report (2022): Bangladesh Bureau of Statistics. 2022 [cited 2023 Oct 28]. https://bbs.portal.gov.bd/sites/default/files/files/bbs.portal.gov.bd/page/b343a8b4_956b_45ca_872f_4cf9b2f1a6e0/2023-09-27-09-50-a3672cdf61961a45347ab8660a3109b6.pdf

[27] Ansink AC, Tolhurst R, Haque R, Saha S, Datta S, van den Broek NR. Cervical cancer in Bangladesh: community perceptions of cervical cancer and cervical cancer screening. Trans R Soc Trop Med Hyg. 2008;102(5):499–505. doi: 10.1016/j.trstmh.2008.01.022 18387643

[28] Hoque MR, Haque E, Karim MR. Cervical cancer in low-income countries: A Bangladeshi perspective. Int J Gynaecol Obstet. 2021;152(1):19–25. doi: 10.1002/ijgo.13400 32989750

[29] Islam RM, Bell RJ, Billah B, Hossain MB, Davis SR. Lack of Understanding of Cervical Cancer and Screening Is the Leading Barrier to Screening Uptake in Women at Midlife in Bangladesh: Population-Based Cross-Sectional Survey. Oncologist. 2015;20(12):1386–92. doi: 10.1634/theoncologist.2015-0235 26590177 PMC4679089

[30] Mahumud RA, Keramat SA, Ormsby GM, Sultana M, Rawal LB, Alam K, et al. Wealth-related inequalities of women’s knowledge of cervical cancer screening and service utilisation in 18 resource-constrained countries: evidence from a pooled decomposition analysis. Int J Equity Health. 2020;19(1):42. doi: 10.1186/s12939-020-01159-7 32216799 PMC7098106

[31] Rashid S, Labani S, Das BC. Knowledge, Awareness and Attitude on HPV, HPV Vaccine and Cervical Cancer among the College Students in India. PLoS One. 2016;11(11):e0166713. doi: 10.1371/journal.pone.0166713 27861611 PMC5115771

[32] Deguara M, Calleja N, England K. Cervical cancer and screening: knowledge, awareness and attitudes of women in Malta. J Prev Med Hyg. 2020;61(4):E584–92. doi: 10.15167/2421-4248/jpmh2020.61.4.1521 33628965 PMC7888396

[33] Honnavar P, Mansoor E, Tulloch C, Udayan U, Cosmello I, Patel P, et al. Cervical Cancer and Human Papillomavirus Awareness among Women in Antigua and Barbuda. Medicina (Kaunas). 2023;59(7):1230. doi: 10.3390/medicina59071230 37512042 PMC10383998

[34] Somera LP, Diaz T, Mummert A, Badowski G, Choi J, Palaganas H, et al. Cervical Cancer and HPV Knowledge and Awareness: An Educational Intervention among College Students in Guam. Asian Pac J Cancer Prev. 2023;24(2):443–9. doi: 10.31557/APJCP.2023.24.2.443 36853291 PMC10162609

[35] Patel H, Sherman SM, Tincello D, Moss EL. Awareness of and attitudes towards cervical cancer prevention among migrant Eastern European women in England. J Med Screen. 2020;27(1):40–7. doi: 10.1177/0969141319869957 31514572

[36] Warner ZC, Reid B, Auguste P, Joseph W, Kepka D, Warner EL. Awareness and Knowledge of HPV, HPV Vaccination, and Cervical Cancer among an Indigenous Caribbean Community. Int J Environ Res Public Health. 2022;19(9):5694. doi: 10.3390/ijerph19095694 35565089 PMC9105034

[37] Boyd JC. Regression Modeling Strategies: With Applications to Linear Models, Logistic Regression, and Survival Analysis. Frank E. Harrell, Jr. New York: Springer-Verlag New York, Inc., 2001, 568 pp., $94.00, hardcover. ISBN 0-387-95232-2. Clinical Chemistry. 2005;51(1):278–9. doi: 10.1373/clinchem.2004.033688

[38] Acito F. Logistic Regression. Predictive Analytics with KNIME: Analytics for Citizen Data Scientists. Cham: Springer Nature Switzerland; 2023. 125–167. doi: 10.1007/978-3-031-45630-5_7

[39] WMA - The World Medical Association-Declaration of Helsinki 1964. n.d. [cited 18 Nov 2023]. https://www.wma.net/what-we-do/medical-ethics/declaration-of-helsinki/doh-jun1964/

[40] Reichheld A, Mukherjee PK, Rahman SM, David KV, Pricilla RA. Prevalence of Cervical Cancer Screening and Awareness among Women in an Urban Community in South India-A Cross Sectional Study. Ann Glob Health. 2020;86(1):30. doi: 10.5334/aogh.2735 32211300 PMC7082824

[41] Kadian L, Gulshan G, Sharma S, Kumari I, Yadav C, Nanda S, et al. A Study on Knowledge and Awareness of Cervical Cancer Among Females of Rural and Urban Areas of Haryana, North India. J Cancer Educ. 2021;36(4):844–9. doi: 10.1007/s13187-020-01712-6 32112367

[42] Dalla V, Panagiotopoulou E-K, Deltsidou A, Kalogeropoulou M, Kostagiolas P, Niakas D, et al. Level of Awareness Regarding Cervical Cancer Among Female Syrian Refugees in Greece. J Cancer Educ. 2022;37(3):717–27. doi: 10.1007/s13187-020-01873-4 32959214

[43] Williams MS, Kenu E, Adanu A, Yalley RA, Lawoe NK, Dotse AS, et al. Awareness and Beliefs About Cervical Cancer, the HPV Vaccine, and Cervical Cancer Screening Among Ghanaian Women with Diverse Education Levels. J Cancer Educ. 2019;34(5):897–903. doi: 10.1007/s13187-018-1392-y 29974412

[44] Abulizi G, Abulimiti T, Li H, Abuduxikuer G, Mijiti P, Zhang S-Q, et al. Knowledge of cervical cancer and Pap smear among Uyghur women from Xinjiang, China. BMC Womens Health. 2018;18(1):21. doi: 10.1186/s12905-018-0512-5 29343254 PMC5773149

[45] Chali K, Oljira D, Sileshi T, Mekonnen T. Knowledge on cervical cancer, attitude toward its screening, and associated factors among reproductive age women in Metu Town, Ilu Aba Bor, South West Ethiopia, 2018: community-based cross-sectional study. Cancer Rep (Hoboken). 2021;4(5):e1382. doi: 10.1002/cnr2.1382 33934571 PMC8552000

[46] Tripathi N, Kadam YR, Dhobale RV, Gore AD. Barriers for early detection of cancer amongst Indian rural women. South Asian J Cancer. 2014;3(2):122–7. doi: 10.4103/2278-330X.130449 24818108 PMC4014643

[47] Choi YJ, Lee HY, An S, Yoon YJ, Oh J. Predictors of Cervical Cancer Screening Awareness and Literacy Among Korean-American Women. J Racial Ethn Health Disparities. 2020;7(1):1–9. doi: 10.1007/s40615-019-00628-2 31410785

