# Supplementary material for: Awareness about cervical cancer and its socio-economic determinants among adults in Bangladesh: Results from a nationwide cross-sectional study
Source: PLoS One. 2025 Jun 10;20(6):e0325712. doi: 10.1371/journal.pone.0325712 (PMC12151404; doi:10.1371/journal.pone.0325712)
Supplement: S1 File — (DOCX) [file pone.0325712.s001.docx]

**Questionnaire**

**NORTH SOUTH UNIVERSITY**

**Study Title:** Awareness about cervical cancer and its socio-economic determinants among adults in Bangladesh: Result from a nationwide cross-sectional study

| 0.1: ID no. |  |
| --- | --- |
| 0.2: Name of the participant |  |
| 0.3: Date of interview |  |

1. **Socio-demographic information**

| 1. Your age (In years) |  |
| --- | --- |
| 1. Your age (In years) | - Female - Male |
| 1. Current marital status | - Living with a spouse - Living without a spouse - Divorced - Separated - Widowed - Prefer not to say |
| 1. Religion | - Muslim - Hindu - Christian - Buddhist - Others |
| 1. Place of residence | - Rural - Semiurban - Urban |
| 1. Educational Status (In completed years) |  |
| 1. Educational Status of your spouse (In completed years) |  |
| 1. Current Occupation | - Private job - Government job - Business person - Housewife - Student - Agriculture - Others (specify)……………………… |
| 1. Current occupation of your spouse | - Private job - Government job - Business person - Housewife - Student - Agriculture - Others (specify)…………………………… |
| 1. Are you a health worker? | - Yes - No |
| 1. Monthly household income (Tk) |  |
| 1. The number of household members |  |
| 1. The total number of children |  |
| 1. The type of the family | - Nuclear - Joint |
| 1. How frequently do you have routine health check-ups? | - Never - Less than 1year interval - 1-2years interval - 2-5 years interval - More than 5 years interval |

1. **knowledge and source of information about cervical cancer**

| **Questions** | **Response of the Participants** | |
| --- | --- | --- |
| 2.1.a Have you ever heard about cervical cancer? | - Yes | - No |
| 2.1.b If yes (2.1.a), from where have you ever heard about cervical cancer? |  |  |
| 1. Physicians/Nurses/Other health care professionals | - Yes | - No |
| 1. Training related to HPV | - Yes | - No |
| 1. Friends | - Yes | - No |
| 1. Family/ Relatives | - Yes | - No |
| 1. School | - Yes | - No |
| 1. Pharmacy | - Yes | - No |
| 1. Advertisement | - Yes | - No |
| 1. Internet/social media | - Yes | - No |
| 1. Mass Media (TV, Radio, Newspaper) | - Yes | - No |
| 1. Others (please specify) |  | |
