# Supplementary material for: Awareness about cervical cancer and its socio-economic determinants among adults in Bangladesh: Results from a nationwide cross-sectional study
Source: PLoS One. 2025 Jun 10;20(6):e0325712. doi: 10.1371/journal.pone.0325712 (PMC12151404; doi:10.1371/journal.pone.0325712)
Supplement: S2 Table — (DOCX) [file pone.0325712.s002.docx]

**Supplementary Table: Factors associated with cervical cancer awareness among participants in univariate logistic regression analysis.**

| **Characteristic** | **COR**^1^ | **95% CI**^1^ | **p-value** |
| --- | --- | --- | --- |
| **Age (years)** | 0.98 | 0.97 to 1.00 | 0.075 |
| **Sex** |  |  |  |
| Female | — | — |  |
| Male | 0.78 | 0.60 to 1.01 | 0.059 |
| **Marital Status** |  |  |  |
| Living with spouse | — | — |  |
| Living without spouse | 0.38 | 0.24 to 0.62 | **<0.001** |
| Others | 0.87 | 0.47 to 1.73 | 0.665 |
| **Residence** |  |  |  |
| Rural | — | — |  |
| Semi-urban | 1.64 | 1.15 to 2.37 | **0.008** |
| Urban | 1.67 | 1.33 to 2.09 | **<0.001** |
| **Years of Education** | 1.16 | 1.13 to 1.19 | **<0.001** |
| **Occupation** |  |  |  |
| Job | — | — |  |
| Business | 0.14 | 0.09 to 0.23 | **<0.001** |
| Housewife | 0.24 | 0.16 to 0.34 | **<0.001** |
| Others | 0.17 | 0.10 to 0.30 | **<0.001** |
| **Health Care Worker** |  |  |  |
| No | — | — |  |
| Yes | 24.9 | 7.94 to 151 | **<0.001** |
| **Monthly household income (BDT)** |  |  |  |
| <=20000 | — | — |  |
| 20001-35000 | 0.91 | 0.69 to 1.20 | 0.494 |
| 35001-50000 | 1.37 | 1.02 to 1.82 | 0.034 |
| >50000 | 2.23 | 1.57 to 3.20 | **<0.001** |
| **Family Type** |  |  |  |
| Nuclear | — | — |  |
| Joint | 0.74 | 0.59 to 0.92 | **0.006** |
| **Routine Health Checkup** |  |  |  |
| Regular | — | — |  |
| Irregular | 0.45 | 0.34 to 0.59 | **<0.001** |
| Never | 0.58 | 0.43 to 0.79 | **<0.001** |

^1^ COR = Crude Odds Ratio, CI = Confidence Interval
